# Present and Future of the Poor-Law Infirmary

**Published:** 1913-05-24

**Authors:** 


### PRESENT AND FUTURE OF THE POOR-LAW INFIRMARY.

### A Poor-Law Pathological Institute.

By H. B. M.

One of the most urgent needs of the Poor-Law system is a Pathological Institute, where bacteriological and pathological work can be undertaken for the whole of the Metropolitan Poor-Law infirmaries at least. Against such an arrangement is usually urged the point that such laboratory work is performed without the aid of the clinical picture. There must, however, be this division of labour. Special work is inevitably deputed to experts in their particular specialty. In the voluntary hospitals the laboratory technician who reports on a tumour or prepares the vaccine seldom sees the patient.

For every infirmary a certain amount of work necessitating bacteriological technique is required; how much is done and how much is left undone depends mainly on the medical superintendent. But sputa have to be examined, vaccines to be prepared, and sections of doubtful growths to be investigated. Usually there is no equipment for such work; the overworked medical officers have no time to undertake the additional duties involving much time and labour; hence in some infirmaries even the examination of the sputa of the phthisical patients, if the examination be undertaken at all, has to be carried out by private associations on the payment of a fee. The amount varies according to the labour expended on the investigation. These investigations are a source of revenue to these private institutes, and the total annual cost of such work to each different Poor-Law authority must vary from about £15 to £50. A return from each infirmary on this point would make interesting reading. The following suggestion is made on the grounds of efficiency and economy. It is not the duty of the Poor Law to patronise and support private associations if the work can be done as efficiently and economically in an institution of their own. Here, again, centralisation is needed. The Metropolitan Asylums Board have an excellently equipped laboratory at Sutton. The Metropolitan Guardians should combine and present a scheme by which, for a yearly sum, all investigations required would be done at this modern laboratory. For the extra work the Metropolitan Asylums Board would probably need the services of another medical laboratory assistant at a cost of about £350 to £400 per annum. Even Borough Councils should utilise these existing resources instead of keeping private associations busy.

Such an appointment would be a whole-time one. The work would be efficiently done, and the laboratory "technician" might even be able to assist in the elucidation of epidemiological and biological questions, the improvement of bacteriological methods, and other studies, by taking advantage of the vast field thus open to his researches.

